# Supplementary material for: Highly sensitive spatial transcriptomics using FISHnCHIPs of multiple co-expressed genes
Source: Nat Commun. 2024 Mar 15;15:2342. doi: 10.1038/s41467-024-46669-y (PMC10943009; doi:10.1038/s41467-024-46669-y)
Supplement: Supplementary file 1 — Supplementary Information [file 41467_2024_46669_MOESM1_ESM.pdf]

Supplementary Figures for

Highly sensitive spatial transcriptomics using FISHnCHIPs of multiple co-expressed genes

Xinrui Zhou[1,2], Wan Yi Seow[1,2], Norbert Ha[1], Teh How Cheng[1], Lingfan Jiang[1], Jeeranan Boonruangkan[1], Jolene Jie Lin Goh[1], Shyam Prabhakar[1], Nigel Chou[1]*, Kok Hao Chen[1]*

[1] Genome Institute of Singapore, Agency for Science, Technology and Research (A*STAR); 60 Biopolis Street, Singapore 138672, Singapore

[2] These authors contributed equally to this work

*Corresponding author. Email: nigel_chou@gis.a-star.edu.sg, chenkh@gis.a-star.edu.sg

Supplementary Fig. 1

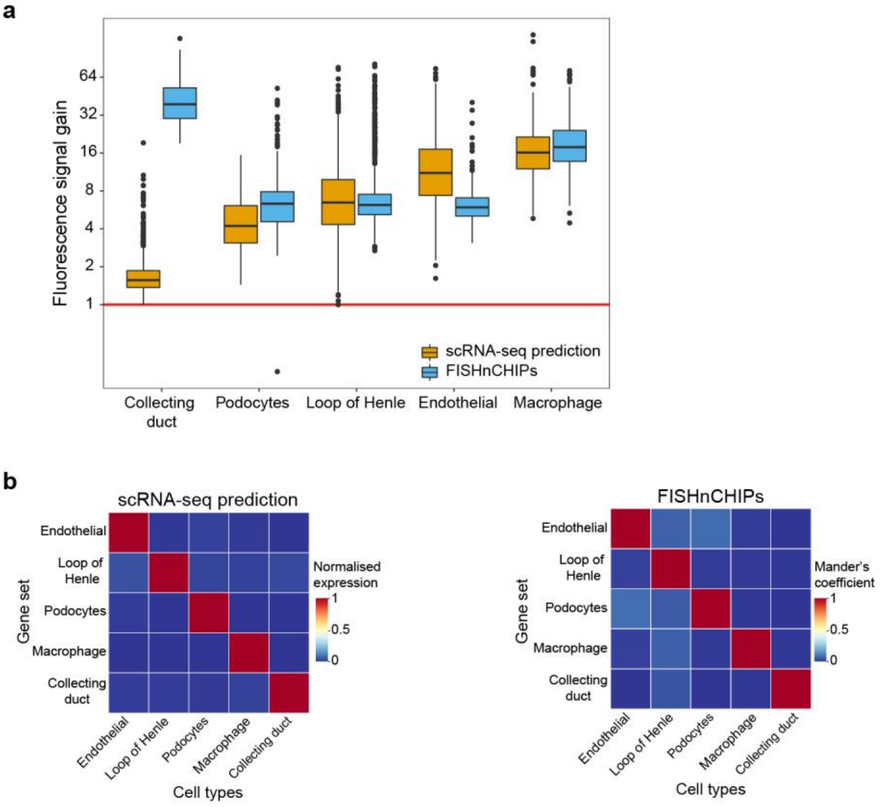

Supplementary Fig. 1 Quantification of FISHnCHIPs (cell-centric) signal and specificity in the mouse kidney (related to Fig. 2).

(**a**) Box plots of the ratio of mean fluorescence intensity per cell of FISHnCHIPs to smFISH (blue), and scRNA-seq predictions: the ratio of counts for 14-23 genes to the top DE gene (yellow). Number of cells (FISHnCHIPs): collecting duct: 146, podocytes: 461, loop of Henle: 727, endothelial: 400, macrophage: 341. Number of cells (scRNA-seq): collecting duct: 1,825, podocytes: 77, loop of Henle: 1,496, endothelial: 701, macrophage: 216. The box plots show the median (center line), the first and third quartiles (box limits), and 1.5× the interquartile range (whiskers). Red line indicates where the fluorescence signal gain is 1. (**b**) Predicted signal crosstalk (left heatmap): Normalized mean scRNA-seq counts for FISHnCHIPs genes across the 5 cell types. Number of cells analyzed is the same as in J. Measured FISHnCHIPs crosstalk (right heatmap): Mander's overlap coefficient across the 5 cell-type channels shown in Fig. 2. Source data are provided as a Source Data file.

Supplementary Fig. 2

a

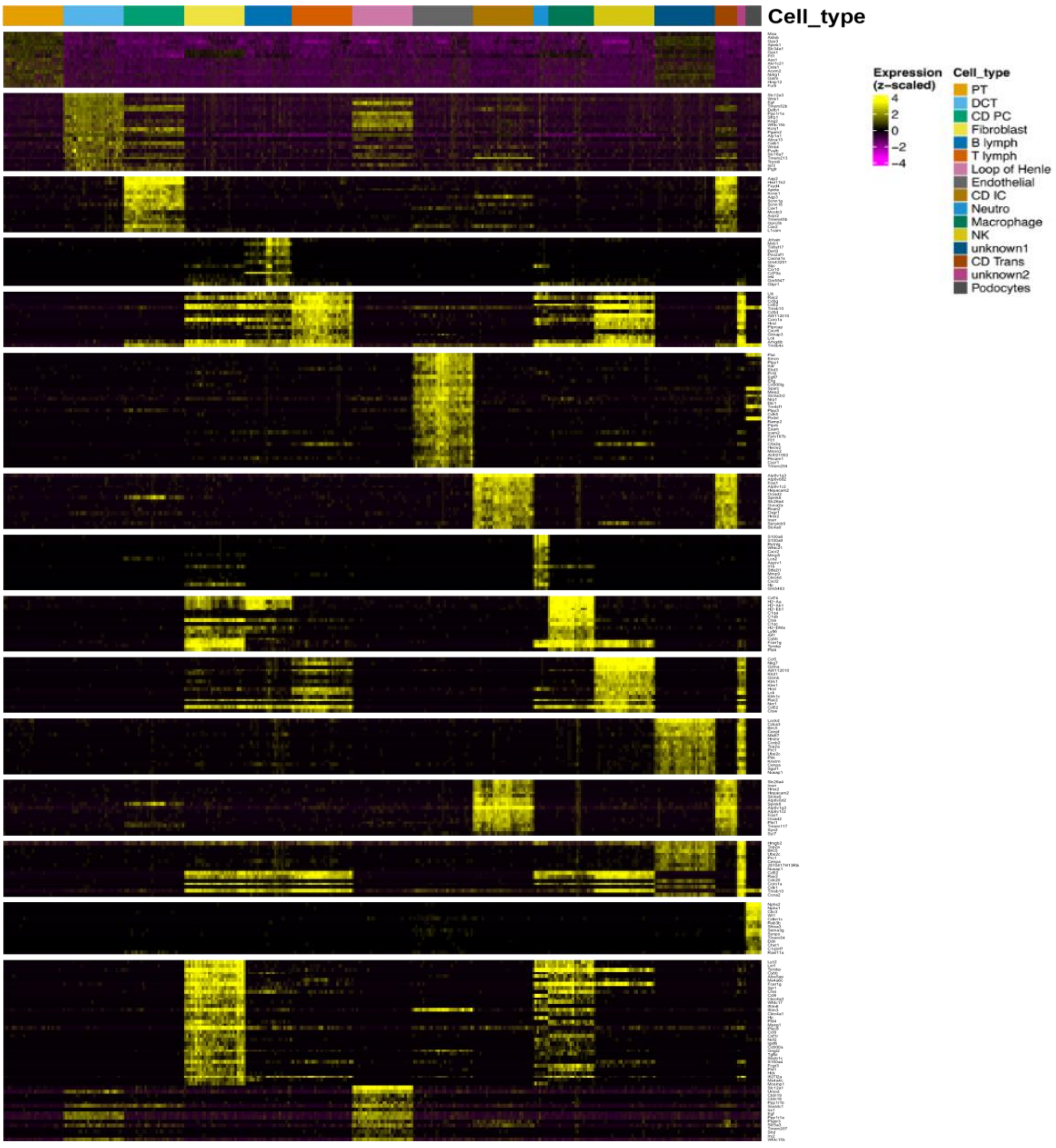

b

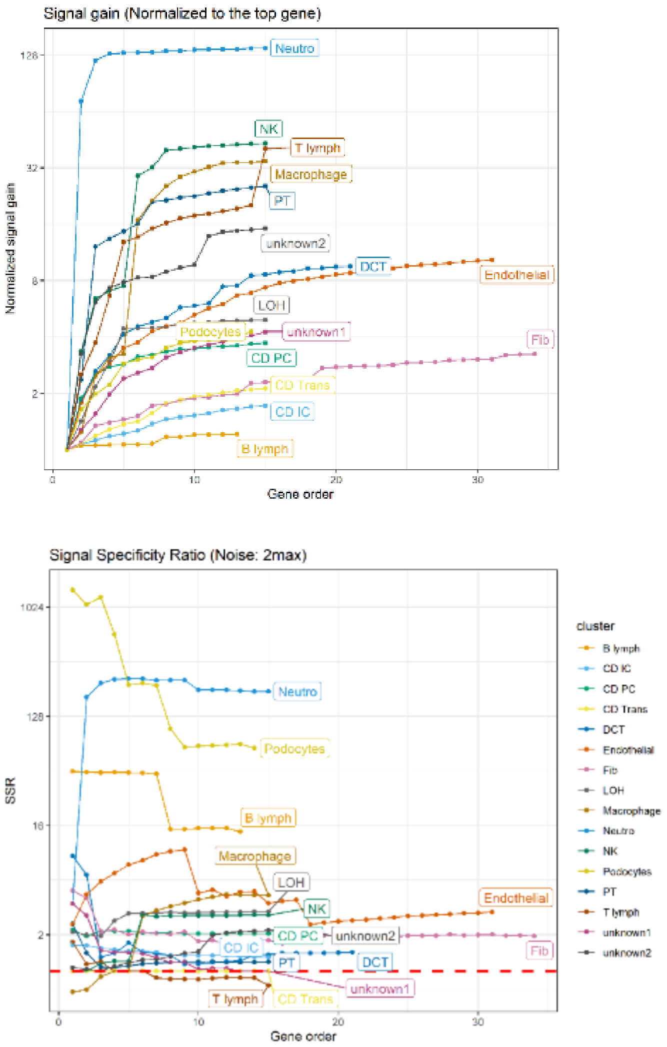
Signal gain (Normalized to the top gene)
Normalized signal gain
Gene order
Neutro
NK
T lymph
Macrophage
PT
unknown2
DCT
Endothelial
LOH
Podocytes
unknown1
Fib
CD PC
CD Trans
CD IC
B lymph
Signal Specificity Ratio (Noise: 2max)
SSR
Gene order
cluster
B lymph
CD IC
CD PC
CD Trans
DCT
Endothelial
Fib
LOH
Macrophage
Neutro
NK
Podocytes
PT
T lymph
unknown1
unknown2

c

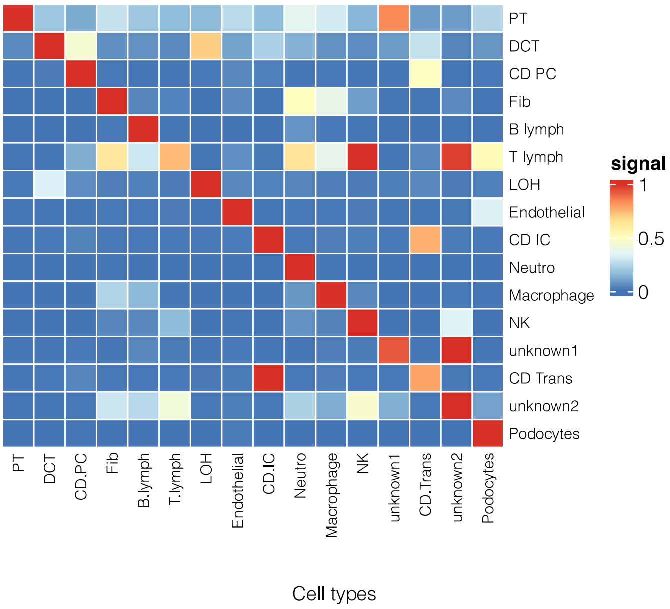
PT
DCT
CD PC
Fib
B lymph
T lymph
LOH
Endothelial
CD IC
Neutro
Macrophage
NK
unknown1
CD Trans
unknown2
Podocytes
signal
1
0.5
0
PT
DCT
CD.PC
Fib
B.lymph
T.lymph
LOH
Endothelial
CD.IC
Neutro
Macrophage
NK
unknown1
CD.Trans
unknown2
Podocytes
Cell types

Supplementary Fig. 2 Computational prediction of FISHnCHIPs (cell-centric) signal gain and specificity.

(**a**) scRNA-seq[27] gene expression heatmap of a FISHnCHIPs gene panel targeting all the previously annotated mouse kidney cell types, sampling a maximum of 300 cells per cluster. (**b**) Predicted Signal Gain (SG) and Signal Specificity Ratio (SSR) as a function of the number of FISHnCHIPs genes. We defined SG as the ratio of the sum of counts for FISHnCHIPs genes to that of the top DE gene, and SSR as the ratio of the sum of counts for FISHnCHIPs genes in the target cell type to that in the most likely off-target cell type. When SSR approaches unity, the fluorescence intensity for the cell type of interest will be equal to an off-target cell type, rendering them indistinguishable. (**c**) Predicted signal crosstalk: Heatmap of the normalized mean scRNA-seq counts of the FISHnCHIPs gene panel across all kidney cell types. Source data are provided as a Source Data file.

Supplementary Fig. 3

a

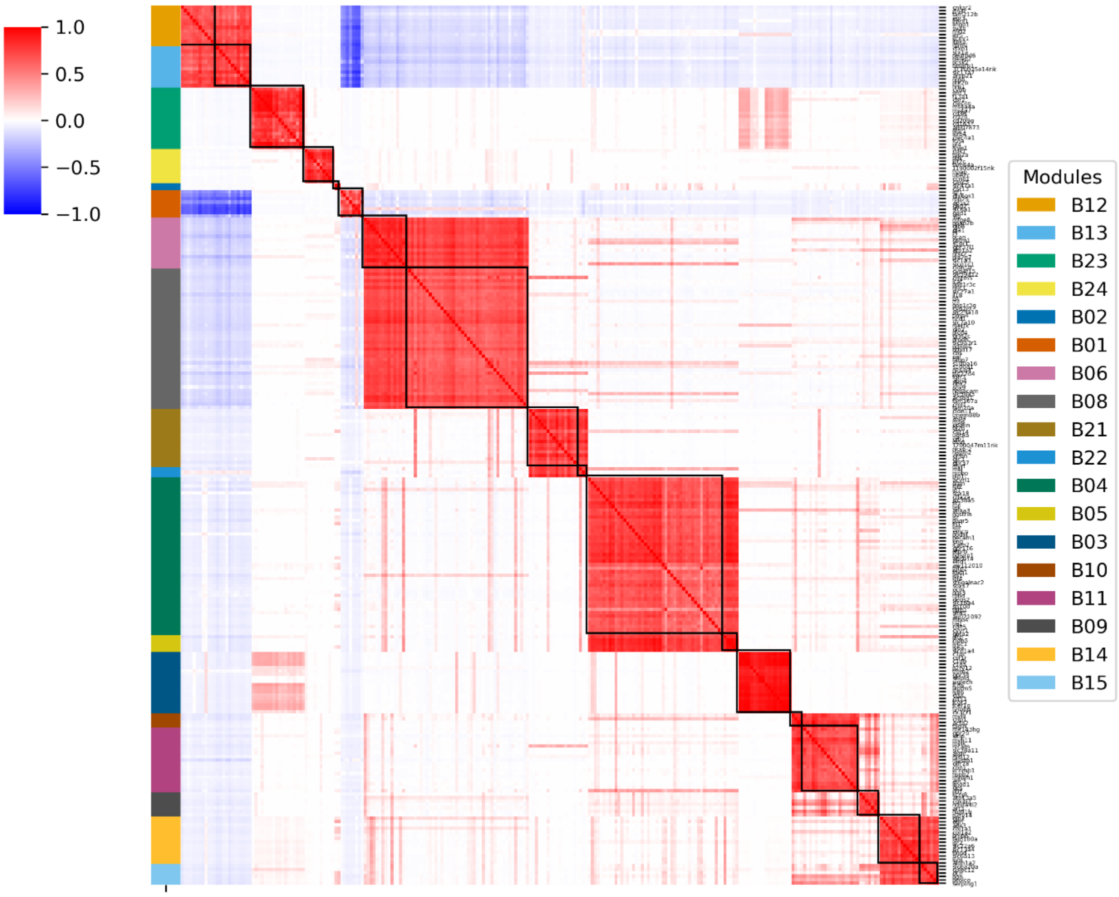

b

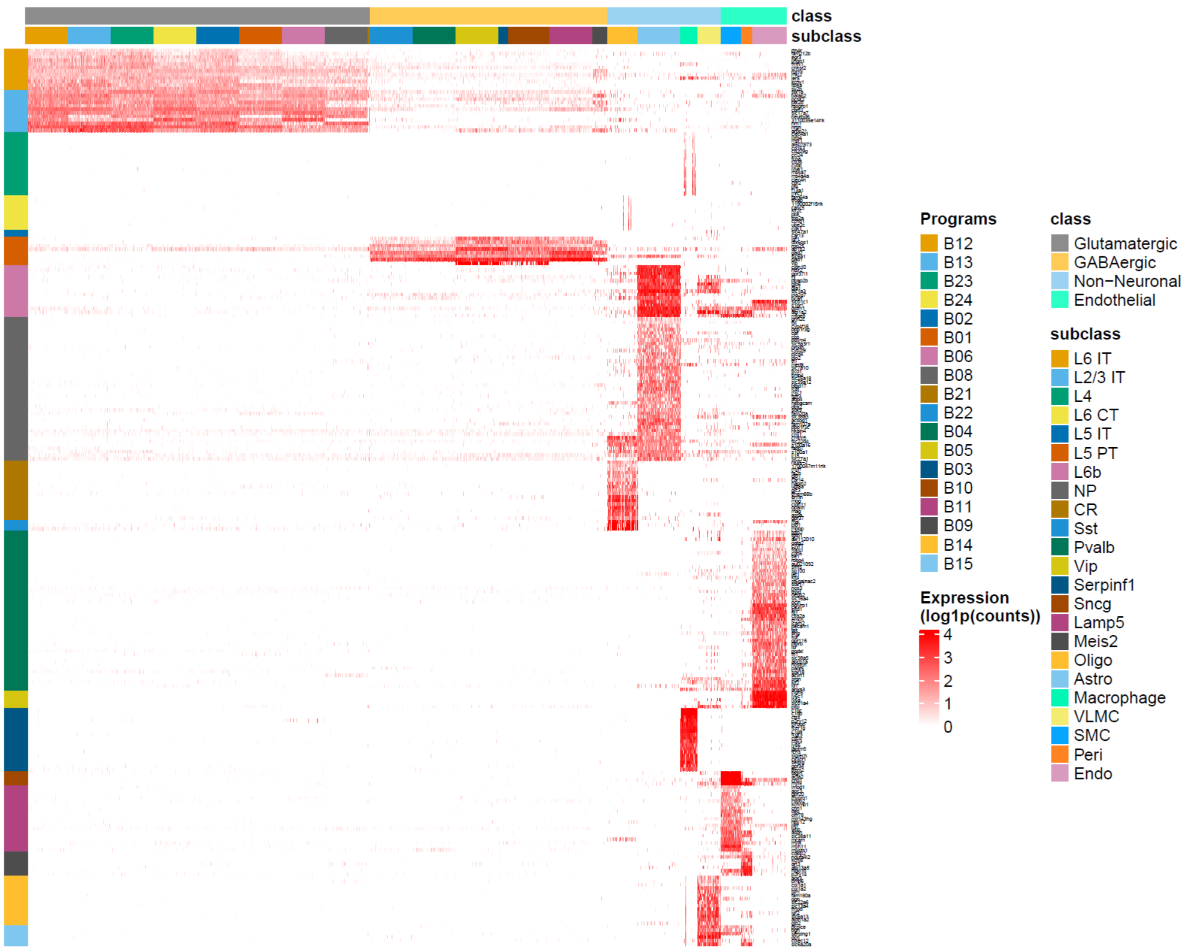

class
subclass
Programs
B12
B13
B23
B24
B02
B01
B06
B08
B21
B22
B04
B05
B03
B10
B11
B09
B14
B15
Expression (log1p(counts))
4
3
2
1
0
class
Glutamatergic
GABAergic
Non−Neuronal
Endothelial
subclass
L6 IT
L2/3 IT
L4
L6 CT
L5 IT
L5 PT
L6b
NP
CR
Sst
Pvalb
Vip
Serpinf1
Sncg
Lamp5
Meis2
Oligo
Astro
Macrophage
VLMC
SMC
Peri
Endo

c

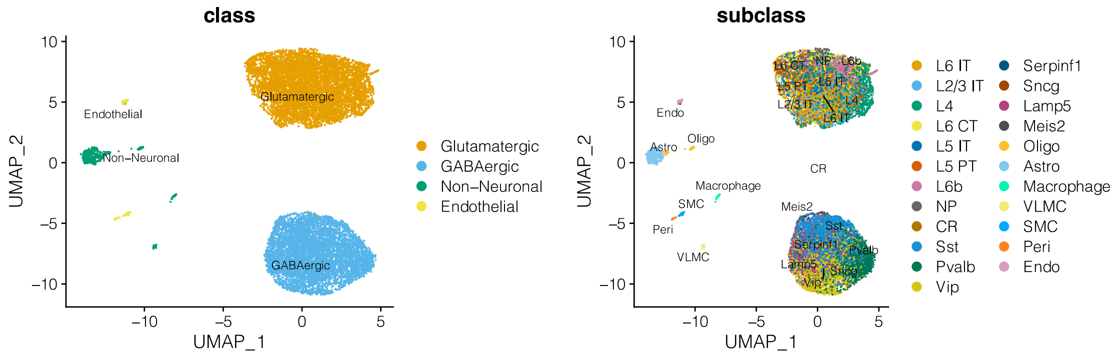

class
Endothelial
Glutamatergic
Non−Neuronal
GABAergic
UMAP_2
UMAP_1
Glutamatergic
GABAergic
Non−Neuronal
Endothelial
subclass
Endo
Oligo
Astro
Macrophage
SMC
Peri
VLMC
CR
Meis2
UMAP_2
UMAP_1
L6 IT
L2/3 IT
L4
L6 CT
L5 IT
L5 PT
L6b
NP
CR
Sst
Pvalb
Vip
Serpinf1
Sncg
Lamp5
Meis2
Oligo
Astro
Macrophage
VLMC
SMC
Peri
Endo

d

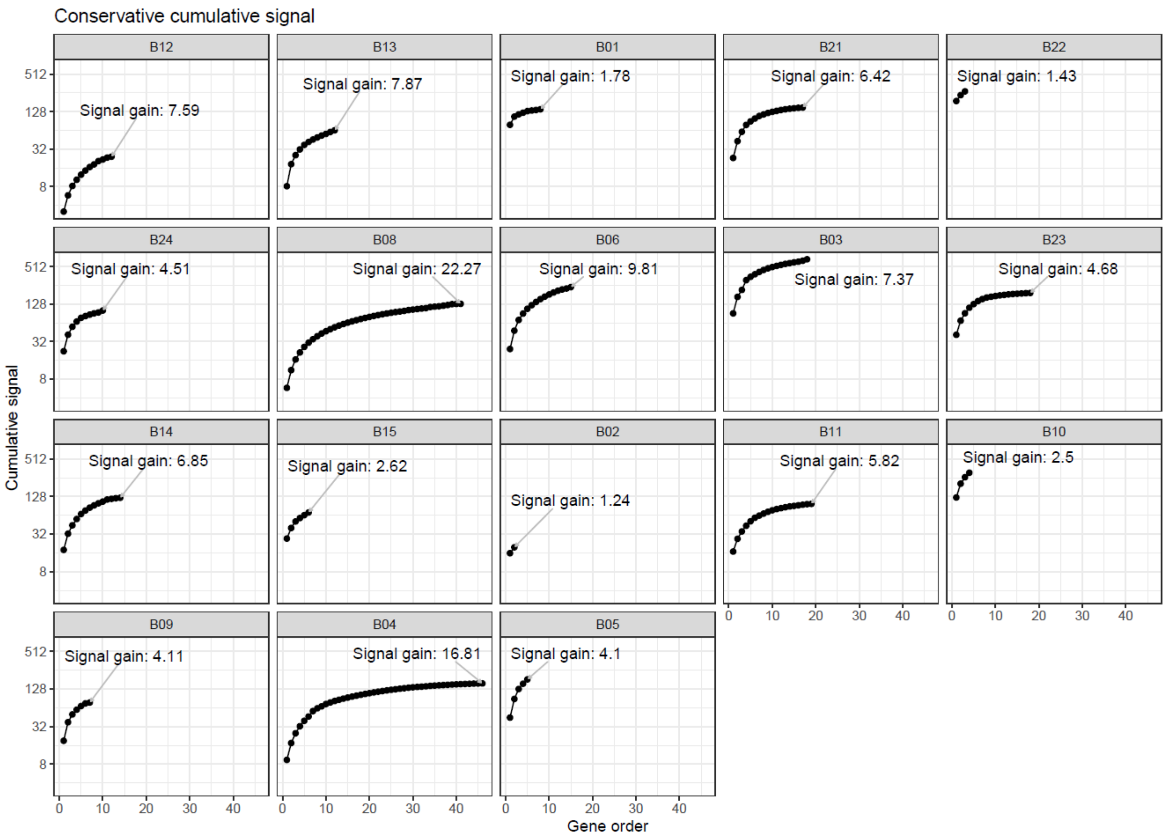
Conservative cumulative signal
B12
Signal gain: 7.59
B13
Signal gain: 7.87
B01
Signal gain: 1.78
B21
Signal gain: 6.42
B22
Signal gain: 1.43
B24
Signal gain: 4.51
B08
Signal gain: 22.27
B06
Signal gain: 9.81
B03
Signal gain: 7.37
B23
Signal gain: 4.68
B14
Signal gain: 6.85
B15
Signal gain: 2.62
B02
Signal gain: 1.24
B11
Signal gain: 5.82
B10
Signal gain: 2.5
B09
Signal gain: 4.11
B04
Signal gain: 16.81
B05
Signal gain: 4.1
Cumulative signal
512
128
32
8
0
10
20
30
40
Gene order

e

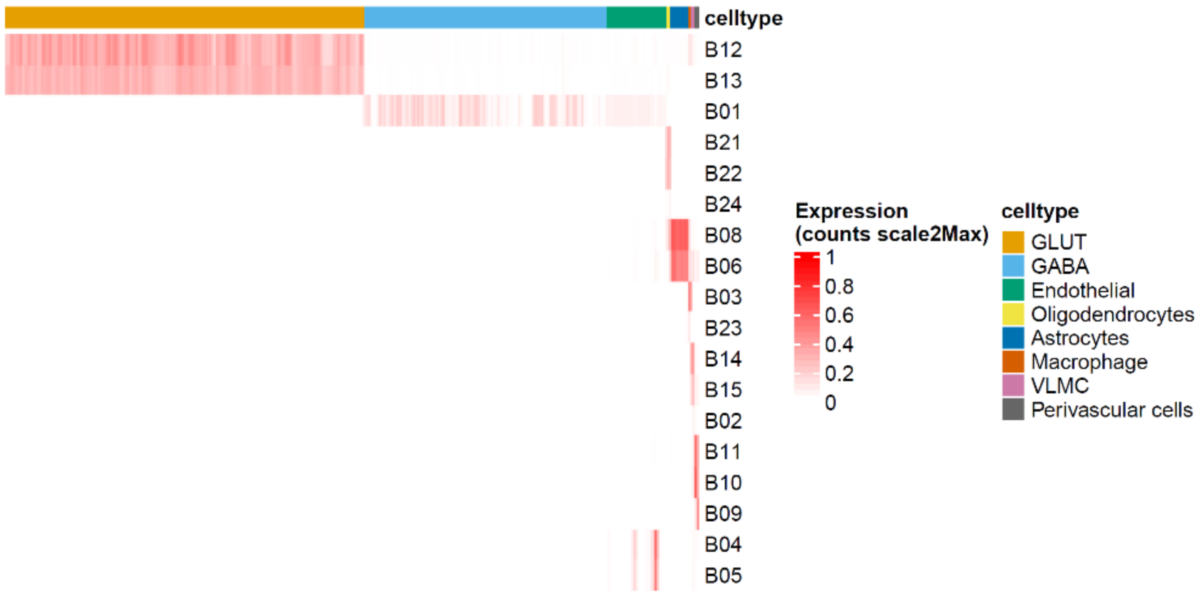
celltype
B12
B13
B01
B21
B22
B24
B08
B06
B03
B23
B14
B15
B02
B11
B10
B09
B04
B05
Expression
(counts scale2Max)
1
0.8
0.6
0.4
0.2
0
celltype
GLUT
GABA
Endothelial
Oligodendrocytes
Astrocytes
Macrophage
VLMC
Perivascular cells

Supplementary Fig. 3 Gene module based FISHnCHIPs library design for the mouse cortex library (imaged in Fig. 3).

(**a**) scRNA-seq[29] gene-gene correlation heatmap (with gene names) for the 255 feature genes in the mouse cortex library (imaged in Fig. 3). We computed their pair-wise Pearson's correlation coefficient and clustered the correlation matrix using the Leiden algorithm. The gene partitions were further sub-clustered using hierarchical clustering into 18 modules. (**b**) scRNA-seq gene expression heatmap for the 255 genes. (**c**) UMAP representation of the predicted clusters from scRNA-seq simulated module-cell (meta-gene) expression, indicated by the labels provided by the scRNA-seq reference dataset. ~8 cell types are clearly separated with the selected features. (**d**) Predicted conservative Signal Gain (cumulative), which is defined as the ratio of the panel signal to the highest gene signal, as a function of the number of genes. (**e**) Predicted module-cell expression heatmap. We grouped the cluster labels into the 8 resolvable cell types (see Supplementary Table 5). Source data are provided as a Source Data file.

Supplementary Fig. 4

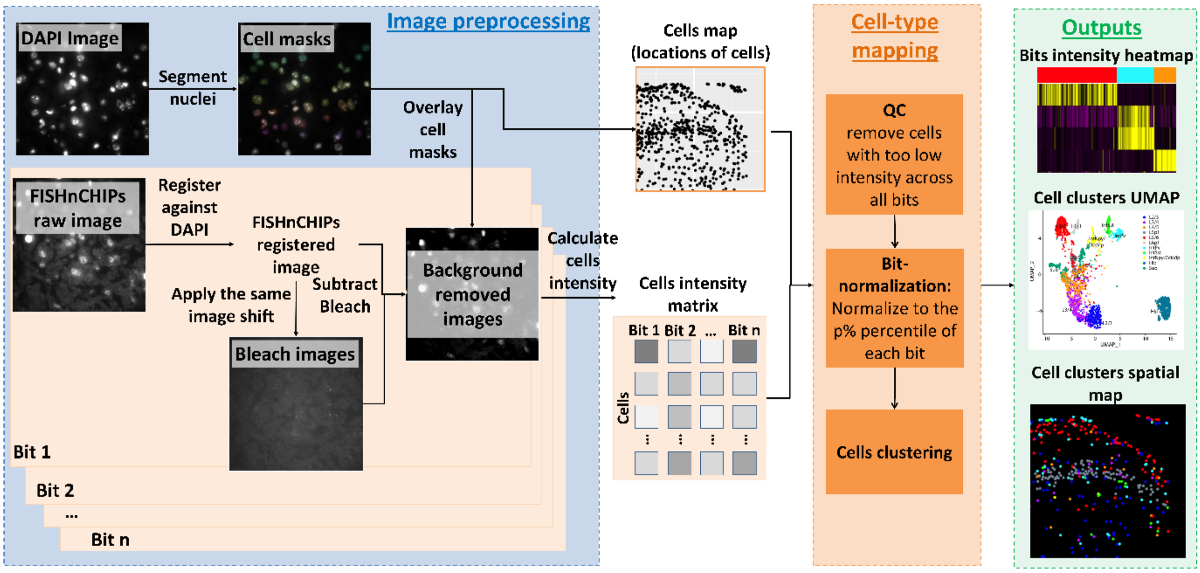

Supplementary Fig. 4 Overview of the FISHnCHIPs image processing and data analysis workflow.

Stepwise data processing (see methods, as well as supplementary software): Inputs are DAPI, FISHnCHIPs, and background (after 55% formamide wash) images. 1) Preprocessing steps include segmentation based on DAPI images to generate cell masks; 2) Registration and background subtraction of FISHnCHIPs images; 3) Cell masks are used to generate cell intensity matrix with a list of cell centroids. 4) Clustering of the cell intensity matrix. 5) Outputs can be visualized in a heatmap, UMAP, or spatial map. Outputs can also be subjected to further analyses, such as classifications of spatial patterns and analysis of cell-cell interactions.

Supplementary Fig. 5

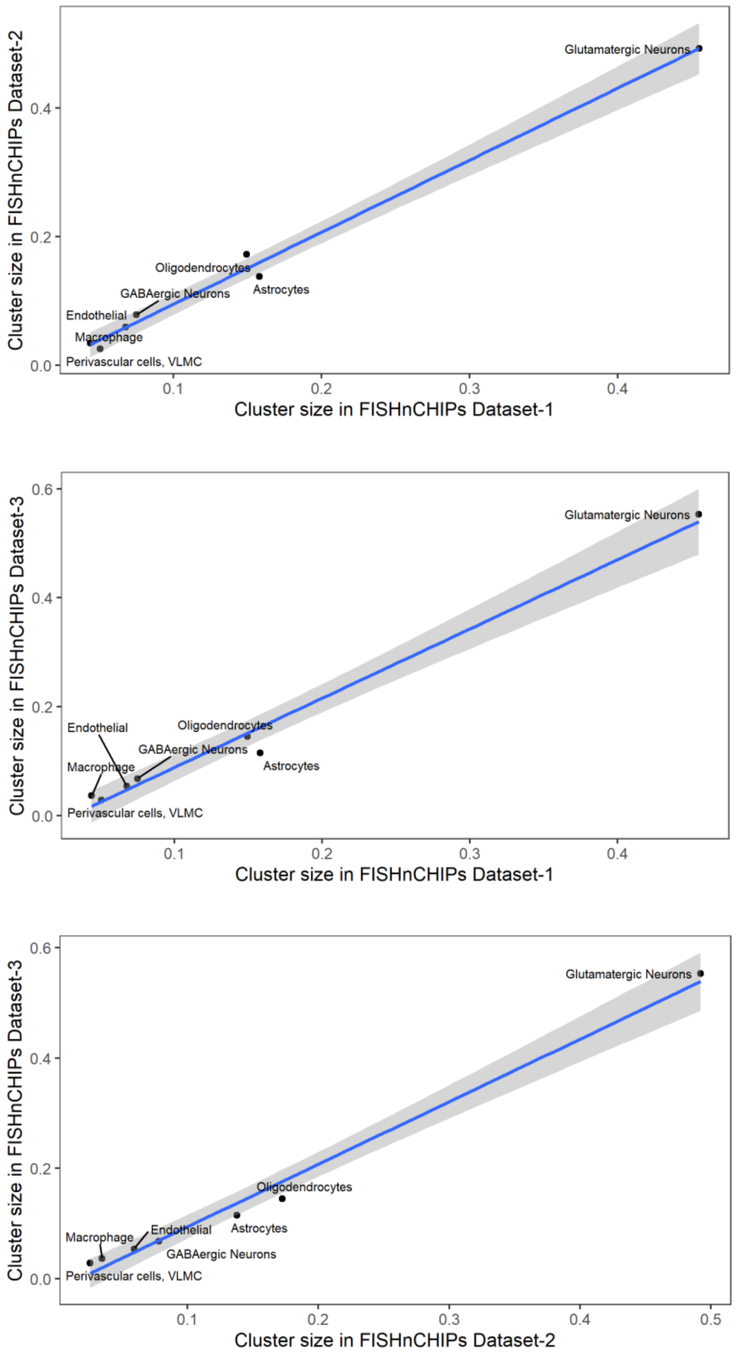

Supplementary Fig. 5 Reproducibility of the mouse brain FISHnCHIPs data (related to Fig. 3) among technical triplicates.

Scatter plots of cell type abundances between technical replicates. Source data are provided as a Source Data file.

Supplementary Fig. 6

a

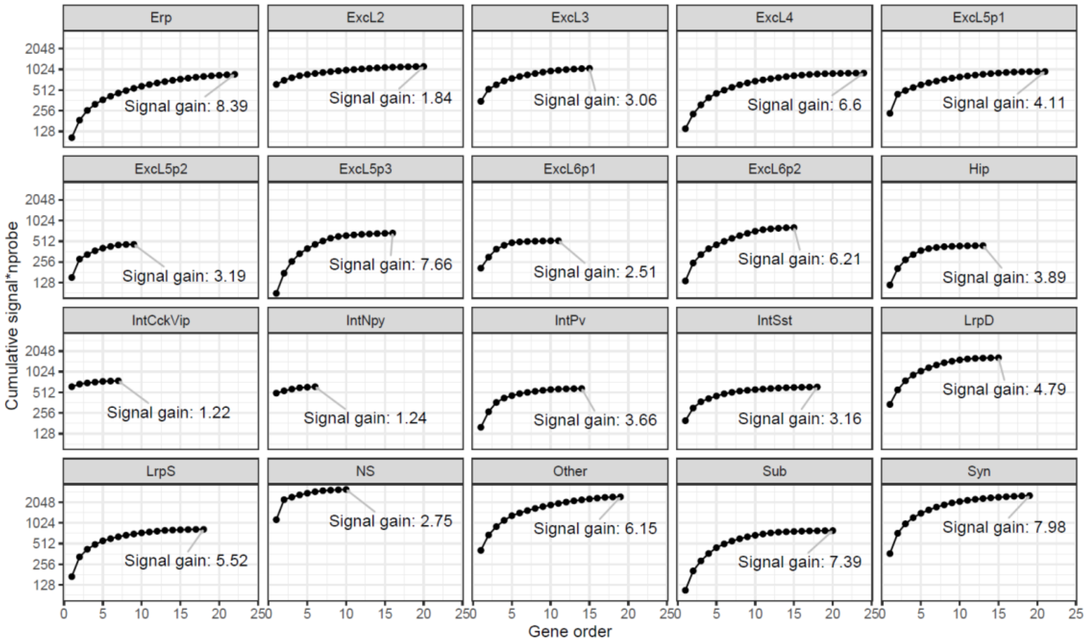

b

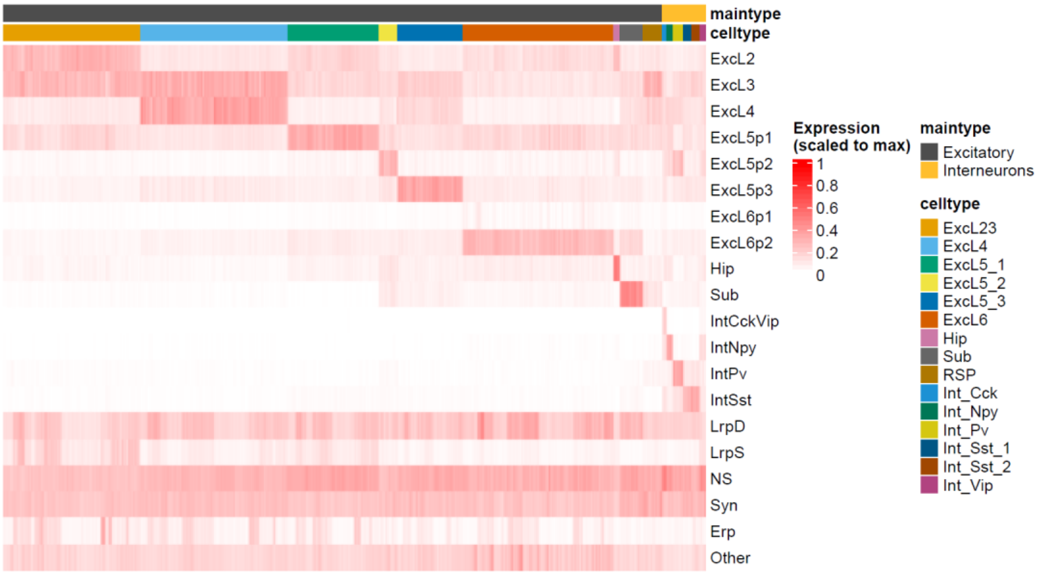

c

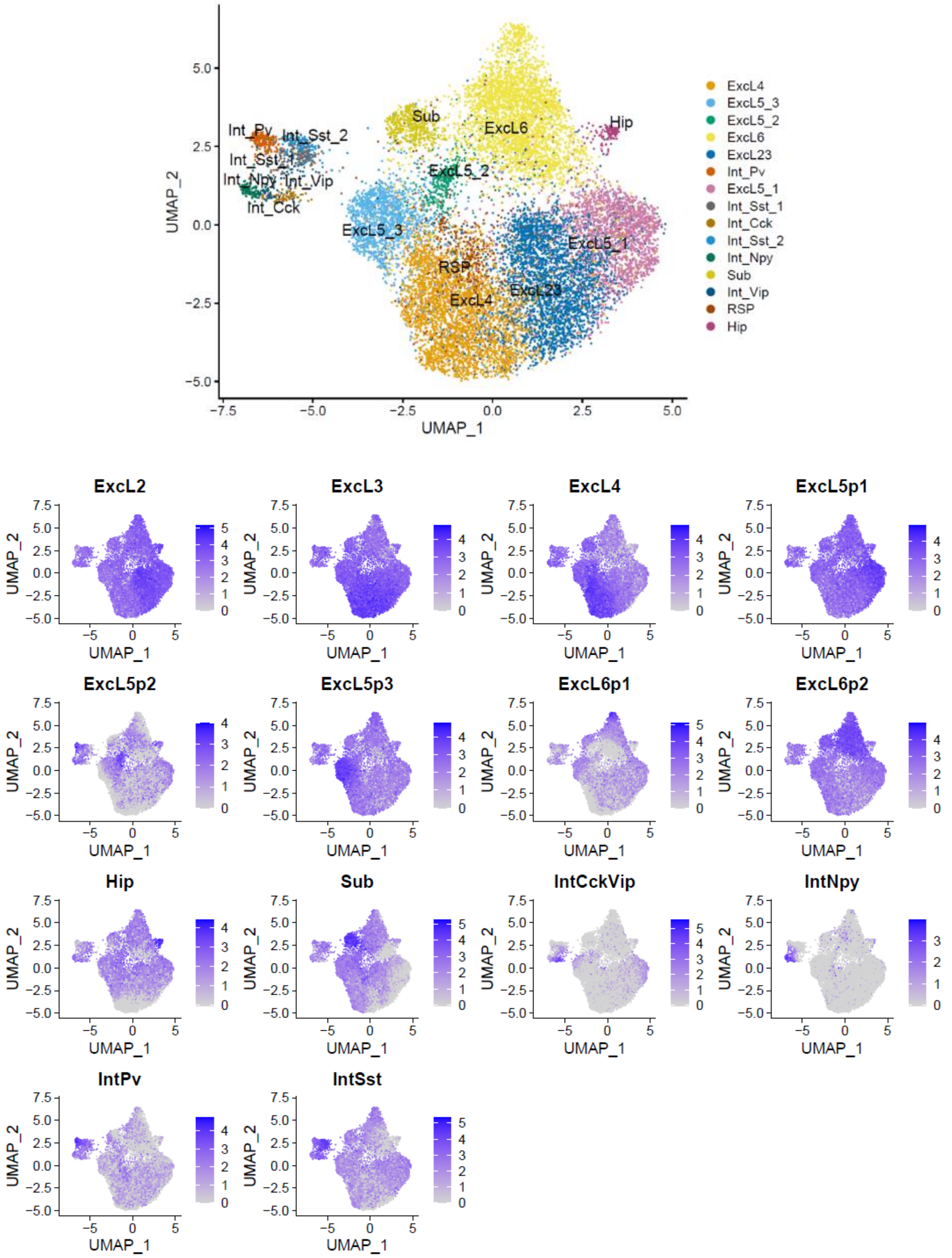

d

Supplementary Fig. 6 Evaluation of the gene expression programs based FISHnCHIPs library for the mouse visual cortex (imaged in Fig. 4) using the scRNA-seq reference dataset. (**a**) Predicted conservative Signal Gain (cumulative), which is defined as the ratio of

the panel signal to the highest gene signal, as a function of the number of genes. (**b**) Predicted Signal Specificity: scRNA-seq expression heatmap for the 20 programs. (**c**) UMAP representation of the predicted clusters from scRNA-seq simulated program-cell (meta-gene) expression, indicated by the labels provided by the scRNA-seq reference dataset[65]. (**d**) Predicted scRNA-seq feature plots of the 14 identify programs. Source data are provided as a Source Data file.

Supplementary Fig. 7

a

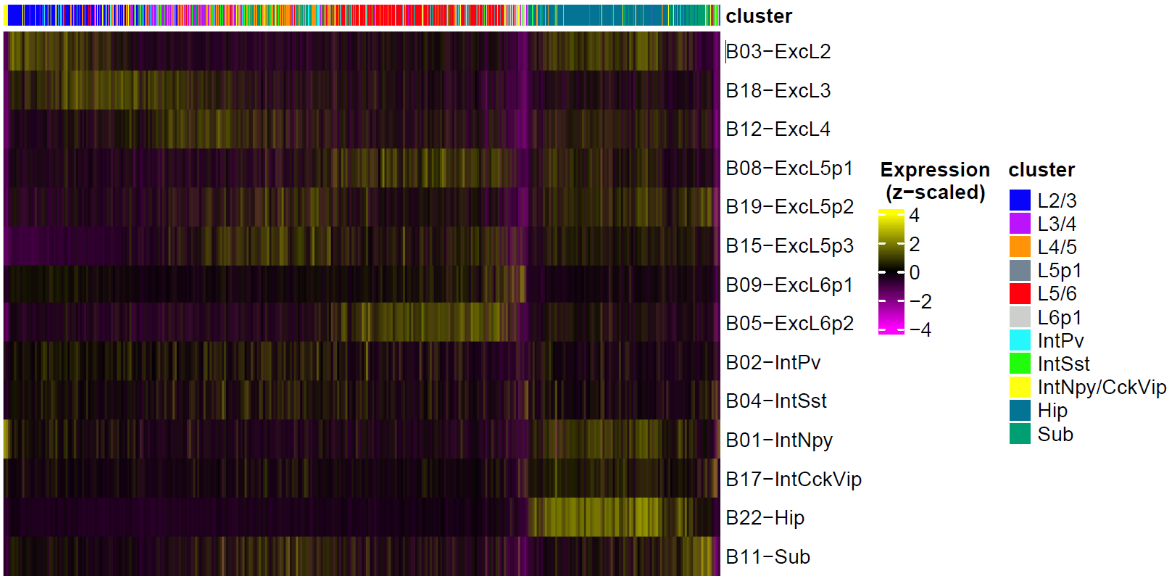

b

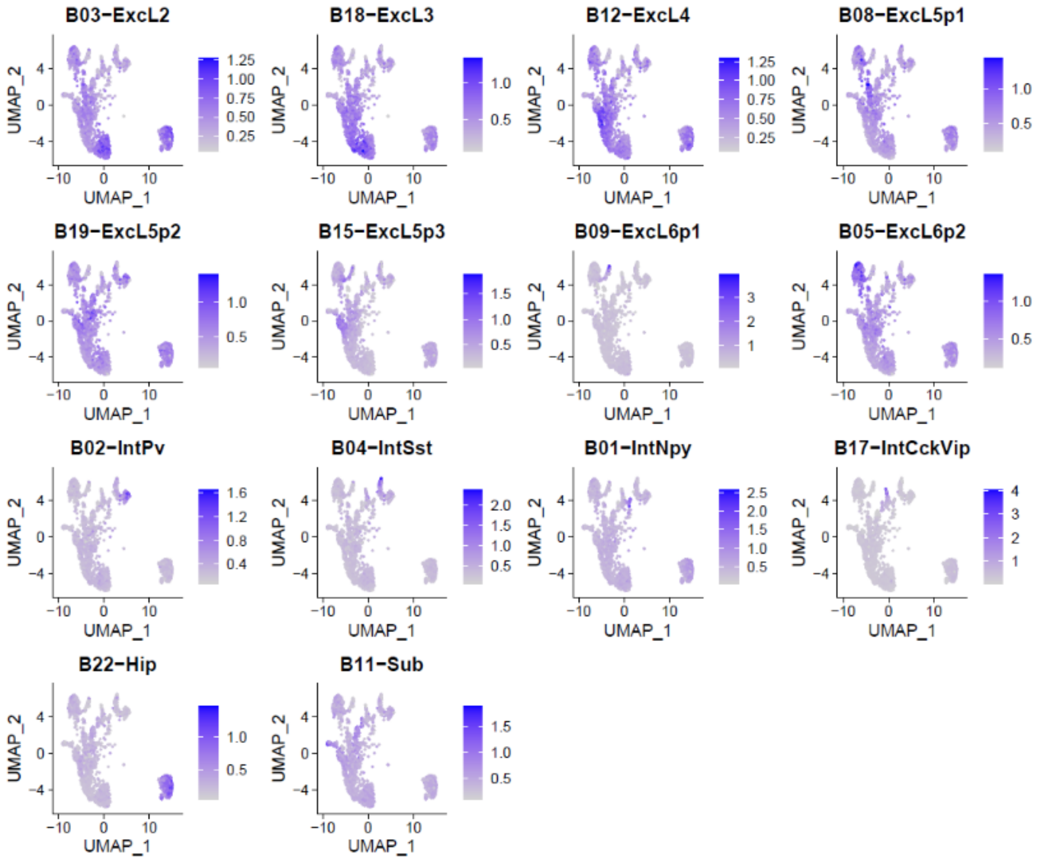

Supplementary Fig. 7 Gradients of gene expression along the cortical depth of the mouse visual cortex as imaged by FISHnCHIPs (related to Fig. 4).

(**a**) FISHnCHIPs expression heatmap of the cell-by-program-intensity matrix (cells are ordered by their distance to the outer edge). (**b**) FISHnCHIPs feature plots of the 14 identity programs.

Supplementary Fig. 8

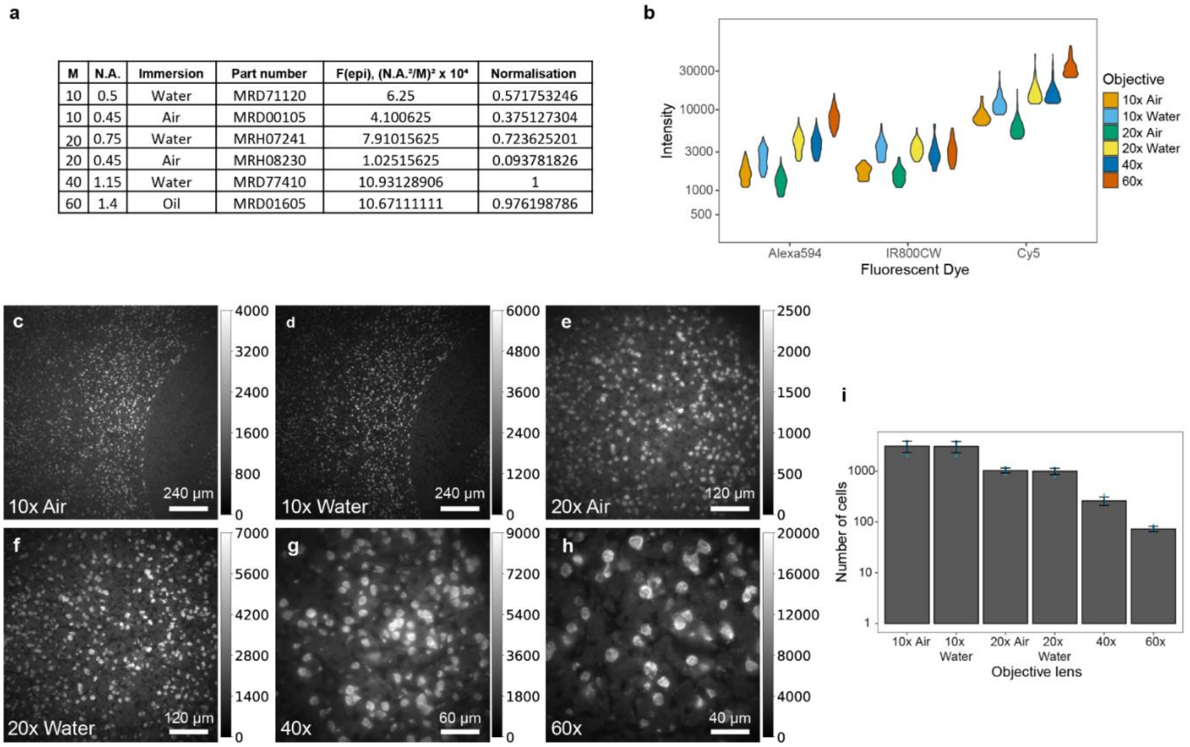

M	N.A.	Immersion	Part number	F(epi), $(N.A.^2/M)^2 \times 10^4$	Normalisation
10	0.5	Water	MRD71120	6.25	0.571753246
10	0.45	Air	MRD00105	4.100625	0.375127304
20	0.75	Water	MRH07241	7.91015625	0.723625201
20	0.45	Air	MRH08230	1.02515625	0.093781826
40	1.15	Water	MRD77410	10.93128906	1
60	1.4	Oil	MRD01605	10.67111111	0.976198786

Supplementary Fig. 8 FISHnCHIPs of the mouse brain imaged under lower magnification.

(**a**) Table of magnification (M), numerical aperture (N.A.), and predicted light gathering power under epi-illumination configuration, F(epi)[66], for 6 different objective lenses. (**b**) Measured mean fluorescence intensity per cell for Alexa594, Cy5, and IR800CW, for 6 different objective lens. (**c-h**) Example unprocessed FISHnCHIPs images (one Field of View, FOV) of the mouse cortex under 6 different objective lenses. Cells labelled with FISHnCHIPs were detected above the background level across all three-color channels, even at the 10x magnification. (**i**) The number of cells detected per FOV (n = 5 FOVs, examined over 1 experiment, error bars indicate the standard deviation). Because of the wider field of view, the number of cells imaged was >~40 fold greater when using the 10x versus 60x objective lenses. Average number of cells: 10x air: 3130, 10x water: 3088, 20x air: 1003, 20x water: 1041, 40x: 261, 60x: 73. We chose the 10x water objectives for Fig. 5 data acquisition. Source data are provided as a Source Data file.

Supplementary Fig. 9

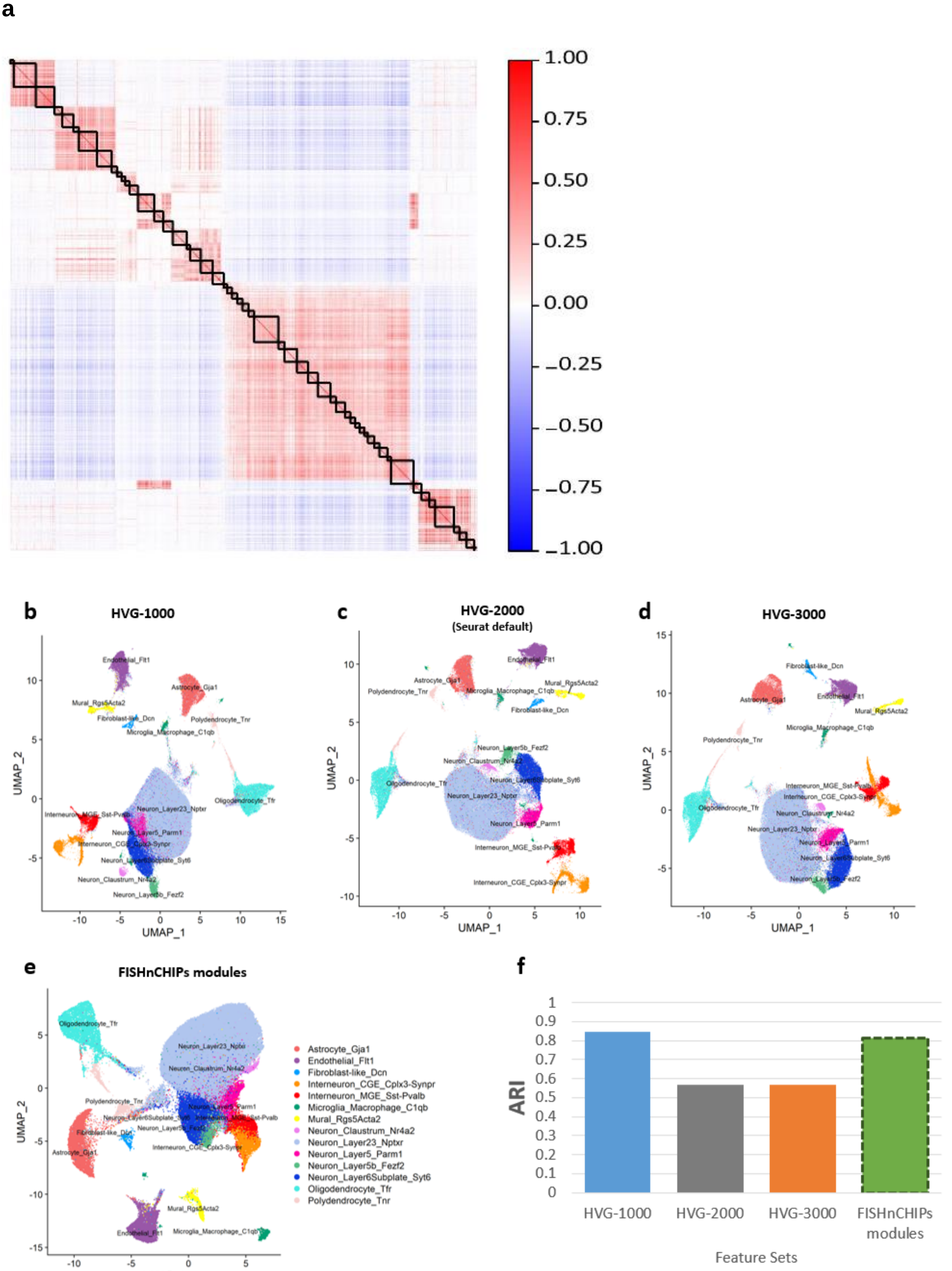

Supplementary Fig. 9 scRNA-seq gene-gene correlation analyses of the FISHnCHIPs genes targeting 53 gene modules in the mouse cortex (imaged in Fig. 5).

(**a**) scRNA-seq gene-gene correlation heatmap for the 674 feature genes in the mouse cortex library (imaged in Fig. 5). We computed their pair-wise Pearson's correlation coefficient and clustered the correlation matrix using the Leiden algorithm. The gene partitions were further sub-clustered using hierarchical clustering into 53 gene modules. (**b-e**) UMAP representation for cells in the scRNA-seq dataset predicted from different feature sets, namely b) 1,000 highly variable genes; c) 2,000 highly variable genes (Seurat default); d) 3,000 highly variable genes; e) 53 modules presented in Fig.5; (**f**) the ARI of clustering cells at a resolution of 0.1 using b-e as features against the labels from the scRNA-seq dataset as ground truth.

Supplementary Fig. 10

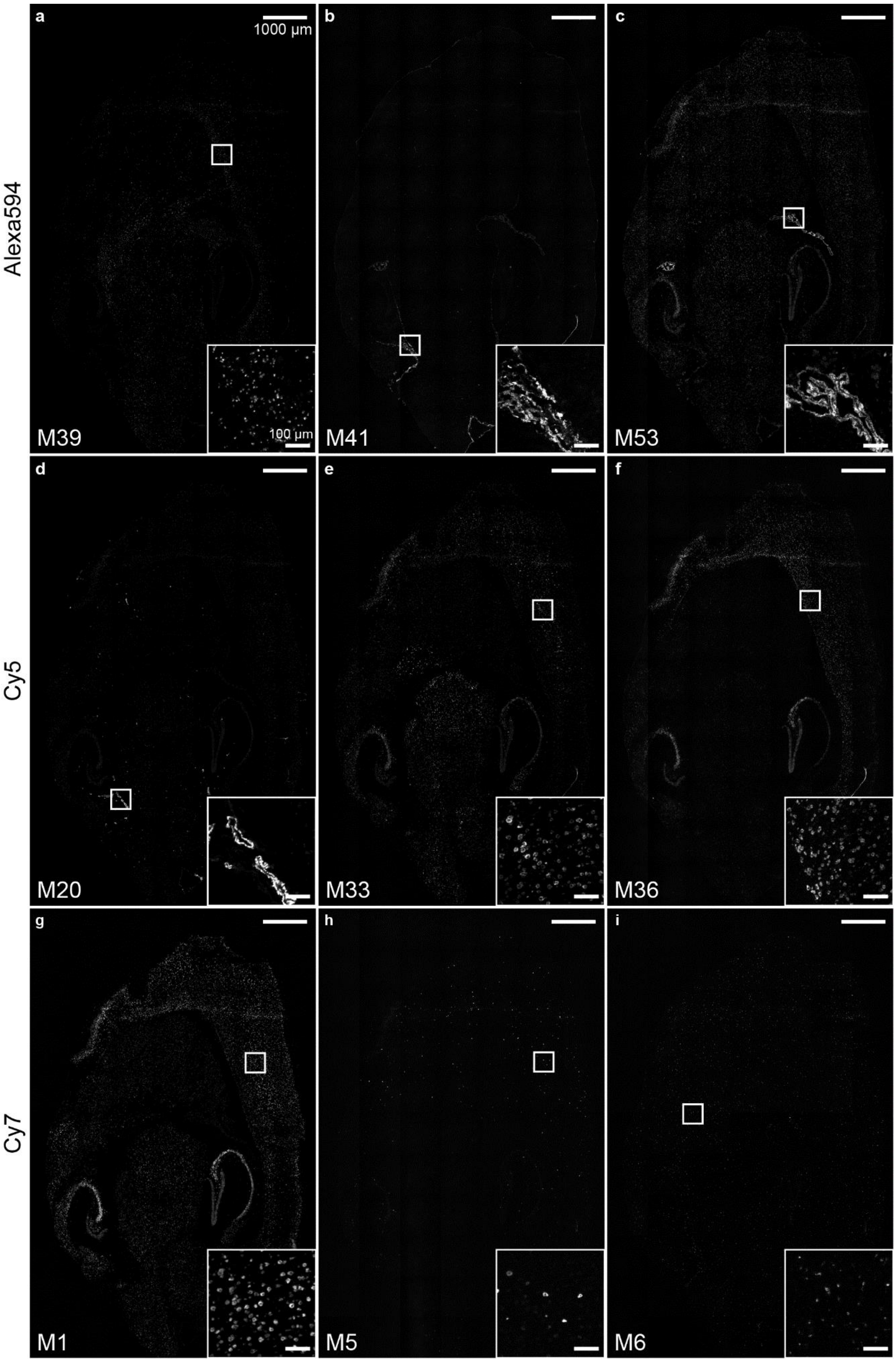

Supplementary Fig. 10 Example normalized images from the 53-modules FISHnCHIPs profiling of gene module 39, gene module 41, gene module 53 using Alexa 594 (**a** - **c**), gene module 20, gene module 33, gene module 36 using Cy5 (**d** - **f**), and gene module 1, gene module 5, and gene module 6 using IRDye 800CW (**g** - **i**) under the 10x objective lens. Scale bar, 1000 µm. Inserts: zoomed in region of the white box. Scale bar, 100 µm.

Supplementary Fig. 11

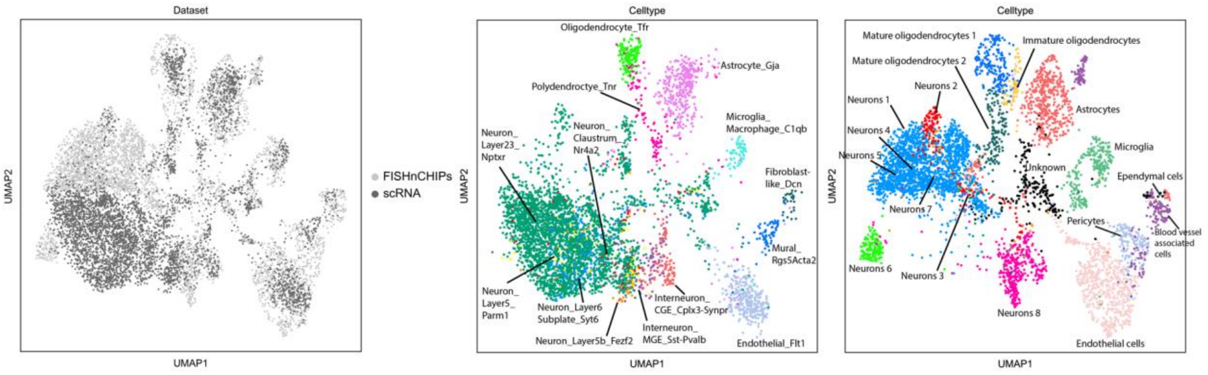

.

Supplementary Fig. 11 FISHnCHIPs and scRNA-seq data integration with Harmony (**a**) UMAP representation for frontal cortex cells from Harmony integration of the scRNA-seq reference and FISHnCHIPs data. (**b**) scRNA-seq cells with cell type labels provided by Saunders *et. al.* [35]. (**c**) FISHnCHIPs cells and labels.

Supplementary Fig. 12

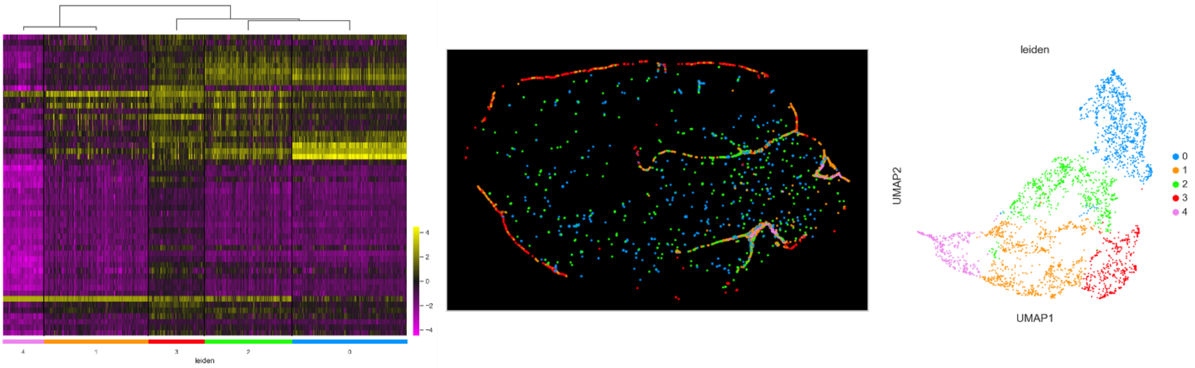

Supplementary Fig. 12 Sub-clustering analyses of the 53-module FISHnCHIPs data revealed subtypes of blood vessel associated and inhibitory cell subtypes with distinct spatial patterns. FISHnCHIPs expression heatmap, spatial map, and UMAP of the subtypes of blood vessel associated cells.

Supplementary Fig. 13

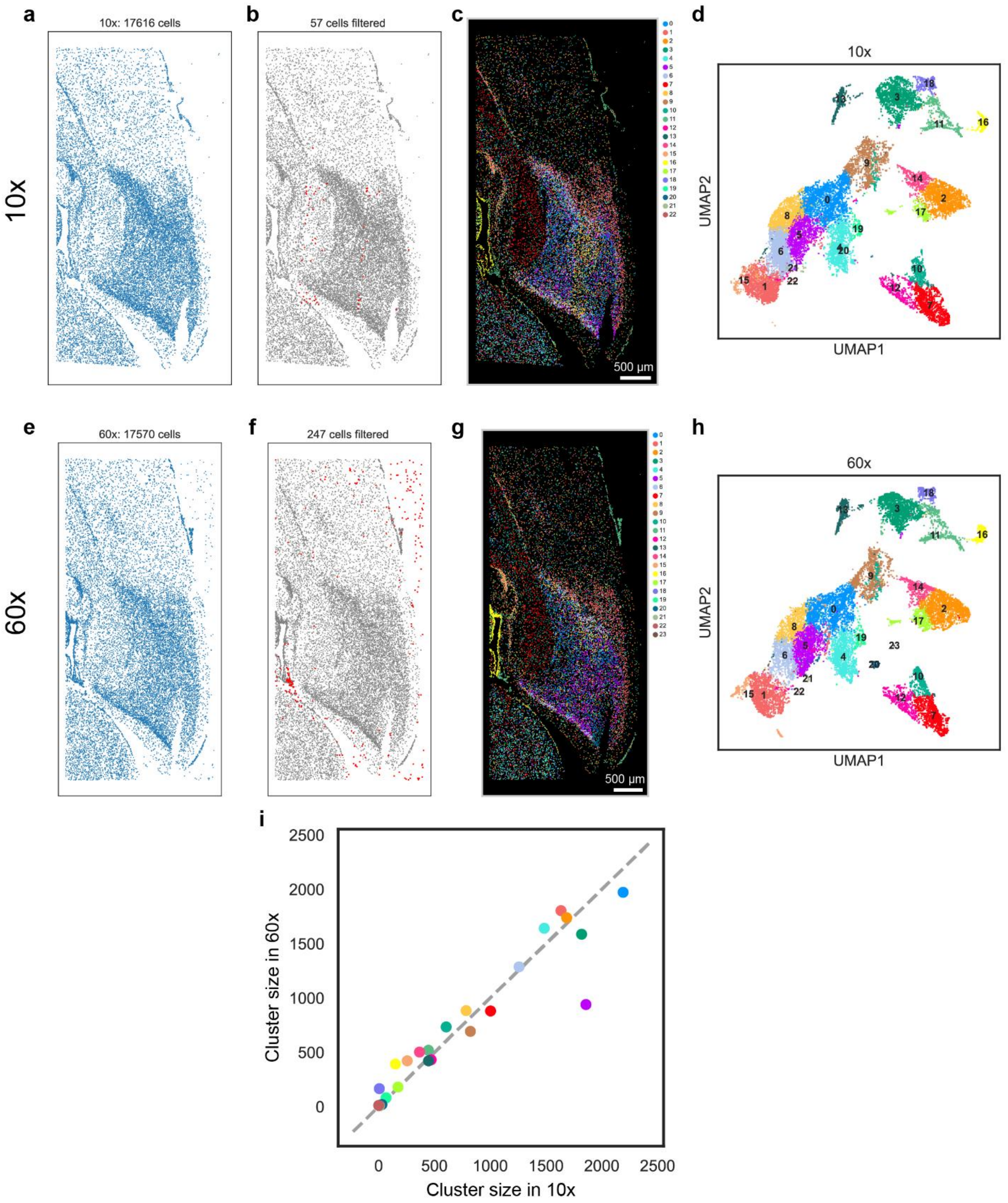

Supplementary Fig. 13 Comparison of the 10x and 60x datasets on adjacent tissue sections. (a, e) Plot of all the segmented cells in the 10x and 60x datasets respectively. (b, f) Cells with low expression across all modules (in red) are filtered out at the first quality control stage. (c, g) Spatial map of cells after Leiden clustering. Scale bar, 500 μm. (d, h)

UMAP representation of the clustering. (i) Scatter plot of number of cells in each cluster detected by 60x versus 10x. Dash line represents the x = y line. Source data are provided as a Source Data file.

Supplementary Fig. 14

Figure	Number of cells from nuclei segmentation	Targeted cell types	Number of cells that passed quality control	Fraction of nuclei imaged that passed quality control
**2**	11,146	Podocytes,<br>Loop of Henle,<br>Endothelial, Collecting Duct,<br>Macrophage	2,075	18.62%
**3**	8,578	Targeted sampling of neurons [29]	6,180	72.04%
**4**	5,552	NMF on neurons only [31]	2,794	50.32%
**5**	56,352	All/untargeted [35]	54,834	97.31%

Supplementary Fig. 14 Comparison of the fraction of cells that passed QC for each library. In the Fig.5 experiment, where we attempted to measure all the cell types, a very high fraction of cells (97.31%) passed QC. In the Fig.4 experiment, where we only targeted the neuronal cells, 50.32% of all cells (determined through DAPI) were quantified as neurons. This number agrees with the Fig.3 data of the cortical region, which also contained ~50% neurons.

Supplementary Fig. 15

a

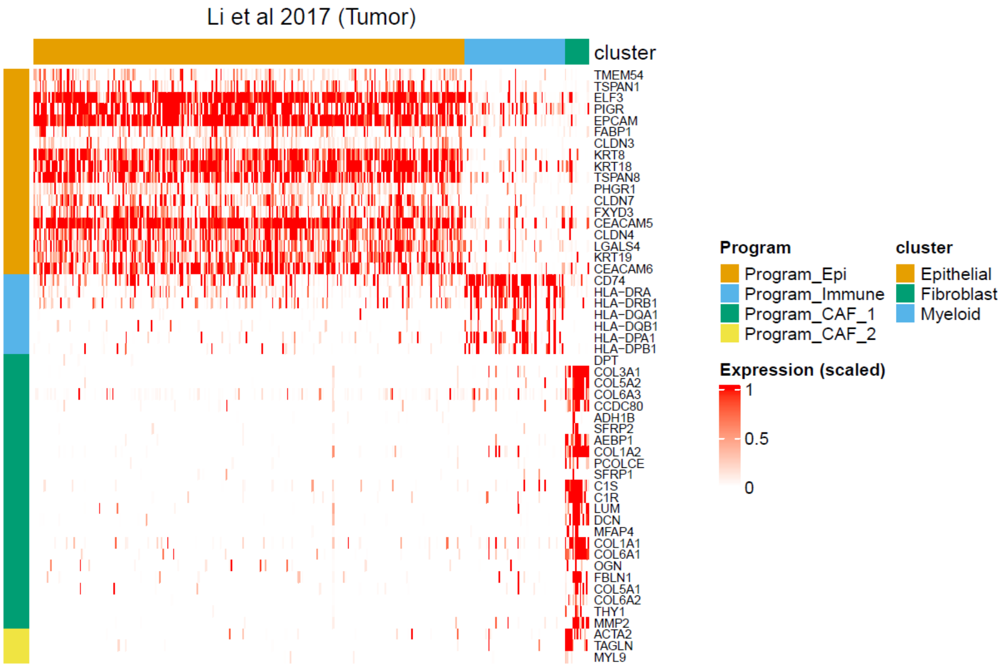

b

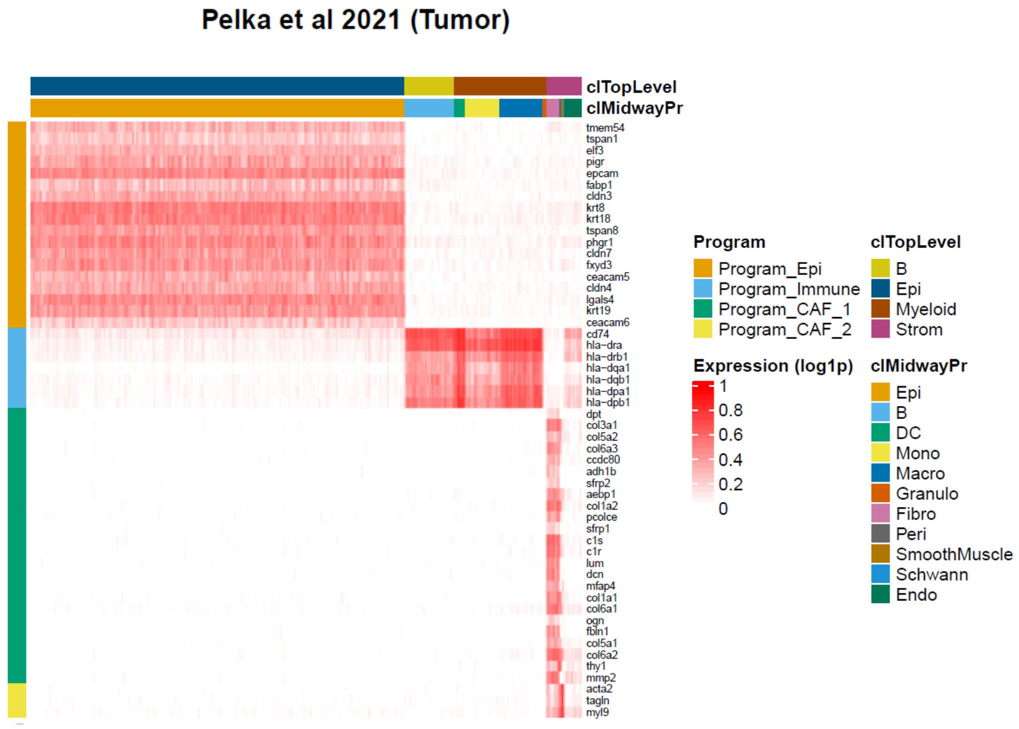

c

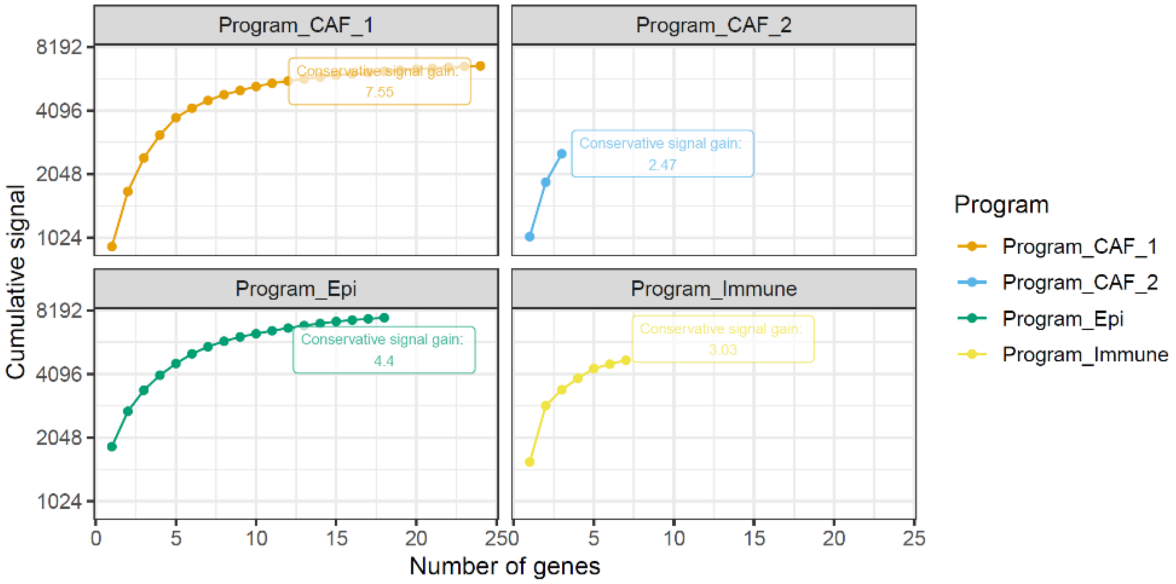

Supplementary Fig. 15 FISHnCHIPs panel for imaging cancer associated fibroblasts (CAFs) subtypes in human colorectal cancer (CRC) tissue (related to Fig. 6).

(**a**) scRNA-seq gene expression heatmap of the human CRC FISHnCHIPs panel[38]. (**b**) scRNA-seq gene expression heatmap of the human CRC FISHnCHIPs panel[53]. (**c**) Predicted conservative signal gain for the human CRC FISHnCHIPs panel. Source data are provided as a Source Data file.

Supplementary Fig. 16

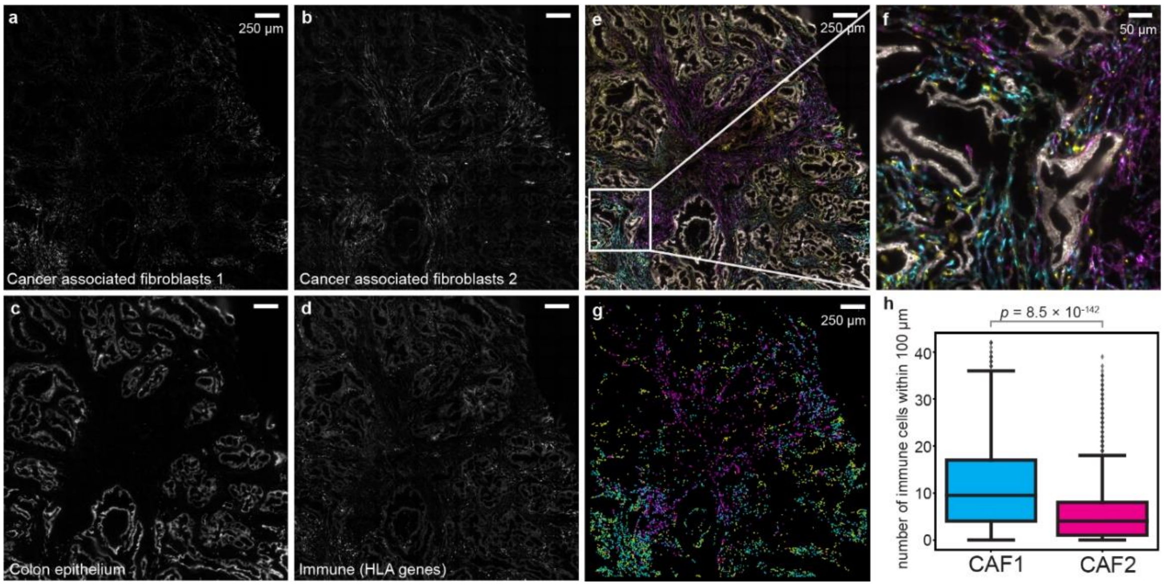

Supplementary Fig. 16 Technical replicate of FISHnCHIPs on human CRC tissue.

(**a**) FISHnCHIPs image of CAF-1 subtype. Scale bar, 250 µm.(**b**) FISHnCHIPs image of CAF-2 subtype. Scale bar, 250 µm. (**c**) FISHnCHIPs image of colon epithelium. Scale bar, 250 µm. (**d**) FISHnCHIPs image of immune (HLA genes). Scale bar, 250 µm. (**e**) Composite FISHnCHIPs image. Scale bar, 250 µm. (**f**) Zoom-in of the white box in E. Scale bar, 50 µm. (**g**) Box plots of the number of immune cells within 100 µm radius of CAF-1 (cyan) and CAF-2 (purple) cells. Immune cells were found 0.51-fold less frequently in the vicinity of CAF-2 than CAF-1. Number of cells, n: CAF-1: 2,548, CAF-2: 2,199, examined over 1 experiment. The box plots show the median (centre line), the first and third quartiles (box limits), and 1.5× the interquartile range (whiskers). $p = 8.5 \times 10^{-142}$, 2-sided Mann-Whitney U test. Source data are provided as a Source Data file.

Supplementary Fig. 17

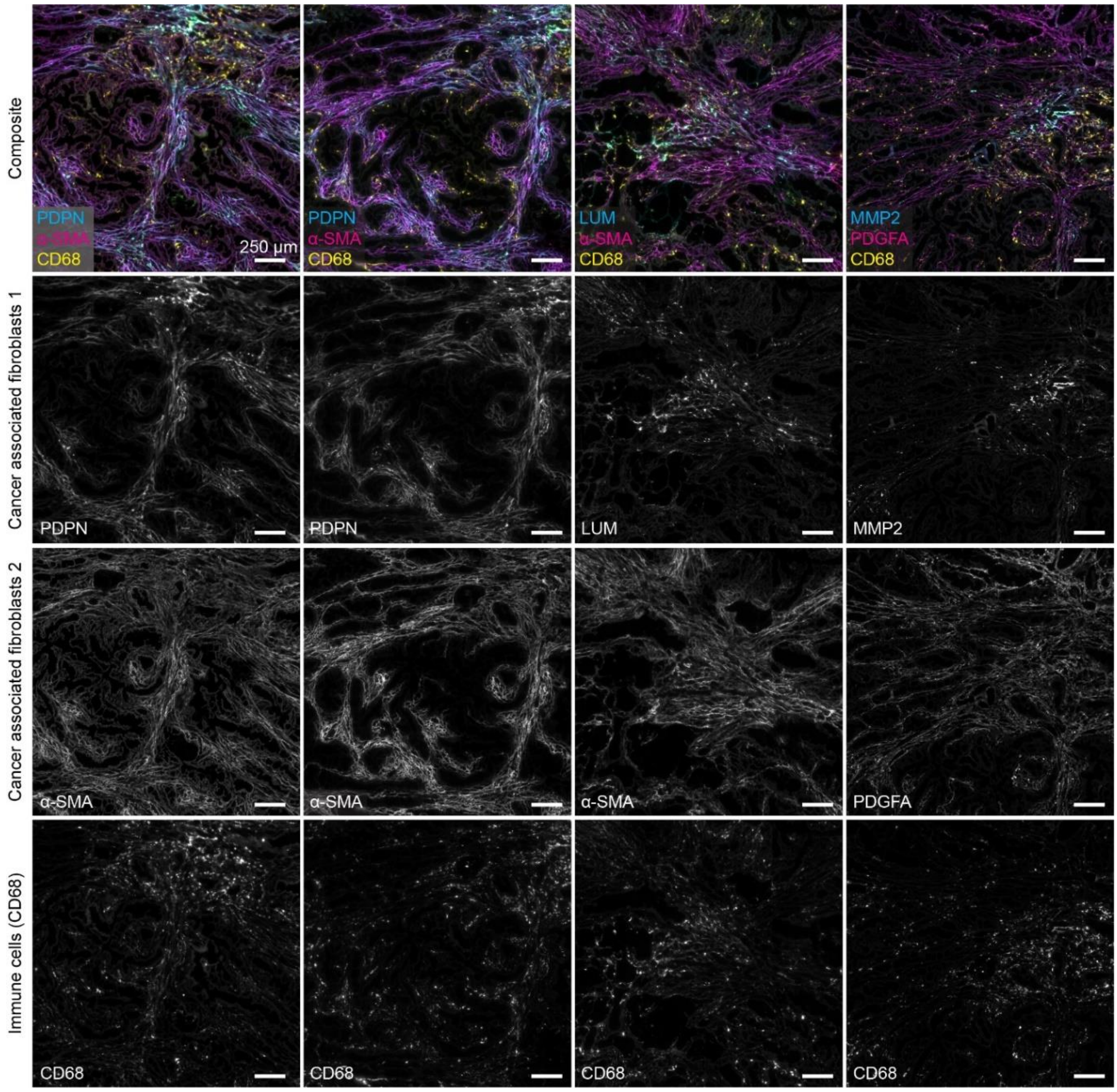

Supplementary Fig. 17 Three-color immunofluorescence staining of the immune marker CD68, CAF-1 markers PDPN, LUM and PDGFA, and CAF-2 markers aSMA and MMP2 on four slices of frozen human colorectal cancer tissue. All images are contrasted at 1 to 99.9 percentiles of the maximum intensity of each channel. Scale bar, 250 µm.

Supplementary Fig. 18

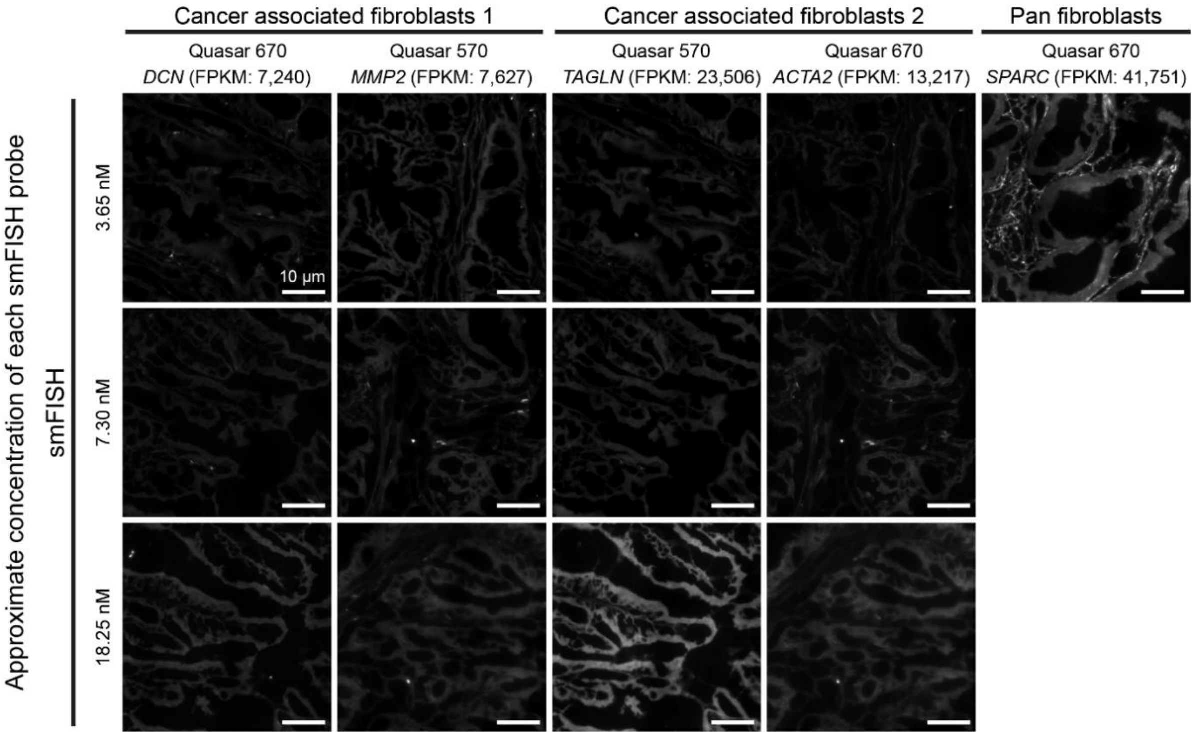

Supplementary Fig. 18. Two-color smFISH staining of the CAF-1 markers *DCN* and *MMP2*, and CAF-2 markers *ACTA2* and *TAGLN* at different concentrations on frozen human colorectal cancer tissue. *DCN* and *TAGLN* are stained together while *MMP2* and *ACTA2* are stained together on the same sample. *SPARC* smFISH staining for pan fibroblast is included as a positive control. Scale bar, 10 µm.

Supplementary Fig. 19

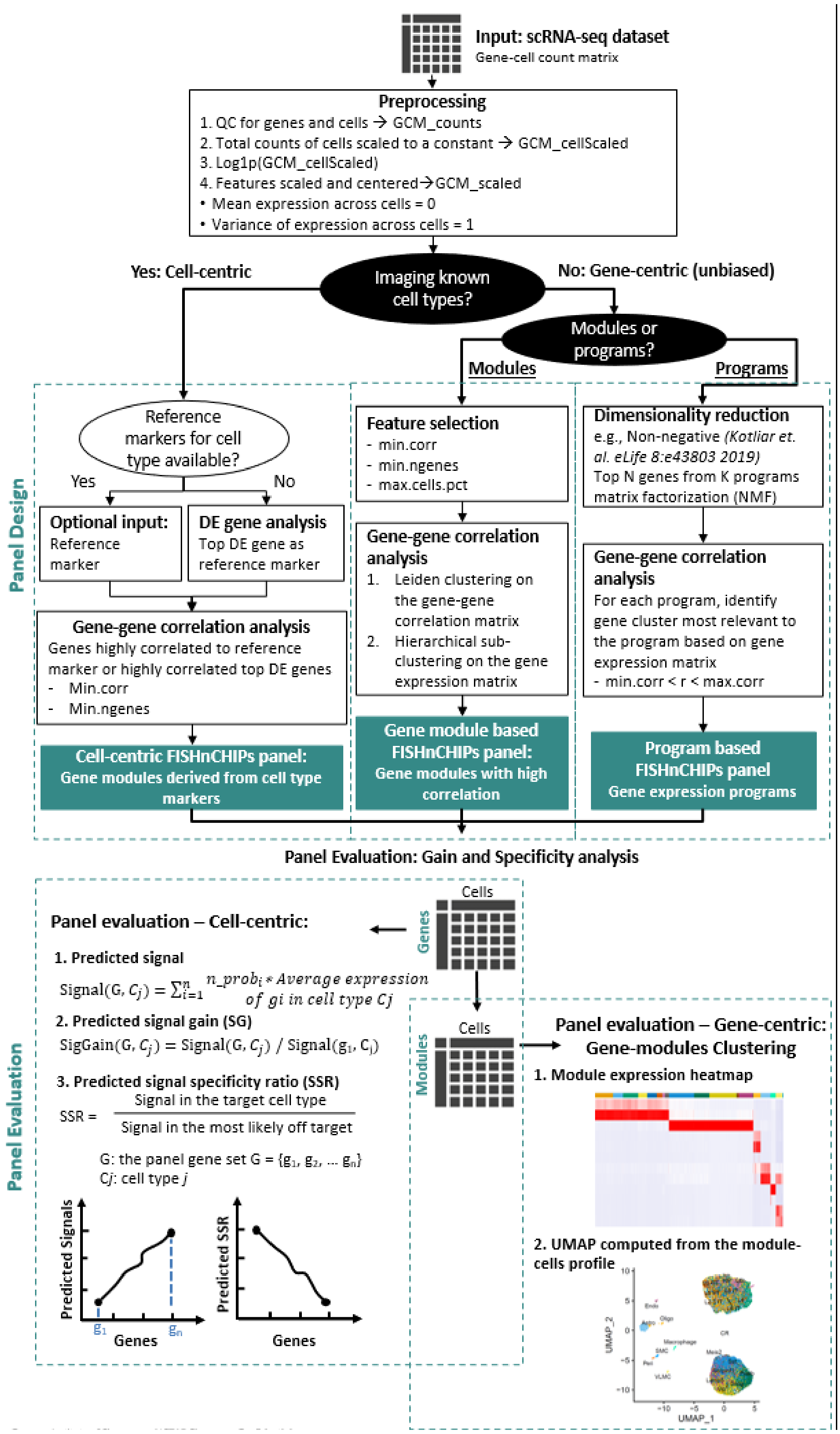

Supplementary Fig.19 FISHnCHIPs panel design software workflow. This flowchart summarizes the FISHnCHIPs panel design process.
