# Supplementary material for: Highly sensitive spatial transcriptomics using FISHnCHIPs of multiple co-expressed genes
Source: Nat Commun. 2024 Mar 15;15:2342. doi: 10.1038/s41467-024-46669-y (PMC10943009; doi:10.1038/s41467-024-46669-y)
Supplement: Supplementary file 3 — Description of Additional Supplementary Files [file 41467_2024_46669_MOESM3_ESM.pdf]

Description of Additional Supplementary Files

File Name: Supplementary Data 1
Description: **FISHnCHIPs libraries and readout probes.** The first column is the gene name, the second column is the transcript ID, and the followings columns include readout ID corresponding to the readout probe sequences. All the probe sequences include the forward and reverse primer sequences used during enzymatic library amplification. Related to Figure 2 to 6. Excel spreadsheets contain 6 tabs: “Fig. 2 probe library”, “Fig. 3 probe library”, “Fig. 4 probe library”, “Fig. 5 probe library”, “Fig. 6 probe library”, and “Readout probes”.

File Name: Supplementary Data 2
Description: **FISHnCHIPs gene panels.** Gene names and Transcript IDs for all the gene panels. Related to Fig. 2 to 5. Excel spreadsheets contain 5 tabs: “Fig. 2 Gene panel”, “Fig. 3 Gene panel”, “Fig. 4 Gene panel”, “Fig. 5 Gene panel”, and “Fig. 6 Gene panel”.

File Name: Supplementary Data 3
Description: **FISHnCHIPs gene modules and GO terms.** For the gene modules generated for the mouse brain, gene ontology enrichment analysis was performed using the web server g:GOSt. To assess the statistical significance of the observed intersections between the genes selected and ontology terms, Fisher’s one-tailed test and multiple testing correction was applied. The term source, name, ID, and adjusted P values are listed in this table. Related to Fig. 3 and 5. Excel spreadsheets contain 2 tabs: “MouseBrain_Fig3GeneModules” and “MouseBrain_Fig5GeneModules”.

File Name: Supplementary Data 4
Description: **Cell type names correspondence between MERFISH dataset and FISHnCHIPs Fig. 3 data.** Each of the cell type annotated in MERFISH is tabulated. Related to Fig. 3. Excel spreadsheets contain 1 tab: “ClusterLabelsMap”.

File Name: Supplementary Data 5
Description: **smFISH probes sequences.** The first column is the target gene name, the second column is the probe sequence, and the third column is the reporter dye. Sequences were purchased from Stellaris RNA FISH probe (LCG Biosearch Technologies). Related to Supplementary Fig. 18. Excel spreadsheets contain 1 tab: “Supp Fig. 18 smFISH probes”.
