# Supplementary material for: Highly sensitive spatial transcriptomics using FISHnCHIPs of multiple co-expressed genes
Source: Nat Commun. 2024 Mar 15;15:2342. doi: 10.1038/s41467-024-46669-y (PMC10943009; doi:10.1038/s41467-024-46669-y)
Supplement: Supplementary file 9 — Reporting Summary [file 41467_2024_46669_MOESM9_ESM.pdf]

nature portfolio

Corresponding author(s): Kok Hao Chen, Nigel Chou

Last updated by author(s): Mar 4, 2024

Reporting Summary

Nature Portfolio wishes to improve the reproducibility of the work that we publish. This form provides structure for consistency and transparency in reporting. For further information on Nature Portfolio policies, see our Editorial Policies and the Editorial Policy Checklist.

Statistics

For all statistical analyses, confirm that the following items are present in the figure legend, table legend, main text, or Methods section.

n/a	Confirmed	
☐	☒	The exact sample size (*n*) for each experimental group/condition, given as a discrete number and unit of measurement
☐	☒	A statement on whether measurements were taken from distinct samples or whether the same sample was measured repeatedly
☐	☒	The statistical test(s) used AND whether they are one- or two-sided<br>*Only common tests should be described solely by name; describe more complex techniques in the Methods section.*
☒	☐	A description of all covariates tested
☒	☐	A description of any assumptions or corrections, such as tests of normality and adjustment for multiple comparisons
☐	☒	A full description of the statistical parameters including central tendency (e.g. means) or other basic estimates (e.g. regression coefficient) AND variation (e.g. standard deviation) or associated estimates of uncertainty (e.g. confidence intervals)
☐	☒	For null hypothesis testing, the test statistic (e.g. *F*, *t*, *r*) with confidence intervals, effect sizes, degrees of freedom and *P* value noted<br>*Give P values as exact values whenever suitable.*
☒	☐	For Bayesian analysis, information on the choice of priors and Markov chain Monte Carlo settings
☒	☐	For hierarchical and complex designs, identification of the appropriate level for tests and full reporting of outcomes
☐	☒	Estimates of effect sizes (e.g. Cohen's *d*, Pearson's *r*), indicating how they were calculated

Our web collection on statistics for biologists contains articles on many of the points above.

Software and code

Policy information about availability of computer code

Data collection	Custom written scripts in Python (https://github.com/ZhuangLab/storm-control) were used to control the instrument collecting the data.
Data analysis	The software to design FISHnCHIPs gene panel and analyze FISHnCHIPs data is available at the following repository: https://github.com/KHChenLab/FISHnCHIPs. It is also available at Zenodo: https://zenodo.org/doi/10.5281/zenodo.10146111.

For manuscripts utilizing custom algorithms or software that are central to the research but not yet described in published literature, software must be made available to editors and reviewers. We strongly encourage code deposition in a community repository (e.g. GitHub). See the Nature Portfolio guidelines for submitting code & software for further information.

Data

Policy information about availability of data

All manuscripts must include a data availability statement. This statement should provide the following information, where applicable:

- Accession codes, unique identifiers, or web links for publicly available datasets
- A description of any restrictions on data availability
- For clinical datasets or third party data, please ensure that the statement adheres to our policy

The FISHnCHIPs expression data and spatial coordinates generated in this study are available as Source Data file. The mouse kidney scRNA-seq dataset used in this study is available in the NCBI Gene Expression Omnibus (GEO) database under accession code GSE107585 [https://www.ncbi.nlm.nih.gov/geo/query/acc.cgi?acc=GSE107585]. The mouse brain scRNA-seq datasets used in this study are available in the NCBI GEO database under accession code GSE115746 [https://

www.ncbi.nlm.nih.gov/geo/query/acc.cgi?acc=GSE115746] and the Dropviz website [http://dropviz.org/]. The human colorectal cancer scRNA-seq datasets used in this study are available in the NCBI GEO database under GSE81861 [https://www.ncbi.nlm.nih.gov/geo/query/acc.cgi?acc=GSE81861] and GSE178341 [https://www.ncbi.nlm.nih.gov/geo/query/acc.cgi?acc=GSE178341].

Research involving human participants, their data, or biological material

Policy information about studies with human participants or human data. See also policy information about sex, gender (identity/presentation), and sexual orientation and race, ethnicity and racism.

Reporting on sex and gender	The aliquot of tumor colon tissue does not contain sex and gender information.
Reporting on race, ethnicity, or other socially relevant groupings	The aliquot of tumor colon tissue does not contain race, ethnicity, or other socially relevant groupings information.
Population characteristics	The aliquot of tumor colon tissue does not contain population characteristics information
Recruitment	To demonstrate the FISHnCHIPs technology, we used an aliquot from a non-individually identifiable tumor colon tissue (A*STAR IRB F-112). The tissue sample was collected by our clinical collaborators, as part of their ongoing research study approved by the institutional review boards of Singhealth (2020-186) for colorectal cancer.
Ethics oversight	This study is approved by the A*STAR Research Integrity, Compliance and Ethics Office for the use of non-individually identifiable human biological materials for in vitro research studies under application number IRB F-112.

Note that full information on the approval of the study protocol must also be provided in the manuscript.

Field-specific reporting

Please select the one below that is the best fit for your research. If you are not sure, read the appropriate sections before making your selection.

☒ Life sciences ☐ Behavioural & social sciences ☐ Ecological, evolutionary & environmental sciences

For a reference copy of the document with all sections, see nature.com/documents/nr-reporting-summary-flat.pdf

Life sciences study design

All studies must disclose on these points even when the disclosure is negative.

Sample size	No statistical methods were used to pre-determine sample size, since the goal was to demonstrate a technology. We chose to use diverse sample types, including mouse kidney, mouse brain, and human colon, to demonstrate the effectiveness of the technology. Fig. 2, 3, 4, 5, and 6 experiments were performed with at least one additional technical replicates with similar results.
Data exclusions	No raw data was excluded from the analyses.
Replication	FISHnCHIPs was demonstrated in multiple tissue types, indicating that the method is robust and reproducible. All results shown are from n = 1 experiment. Fig. 2, 3, 4, 5, and 6 experiments were repeated at least once independently with similar results. All supplementary figures experiments (except for supplementary fig. 8, 13, 17 and 18) were repeated at least once independently with similar results.
Randomization	There is no experiment group in this study and hence no randomization is needed.
Blinding	There is no experimental group in this study and hence no blinding is needed.

Reporting for specific materials, systems and methods

We require information from authors about some types of materials, experimental systems and methods used in many studies. Here, indicate whether each material, system or method listed is relevant to your study. If you are not sure if a list item applies to your research, read the appropriate section before selecting a response.

Materials & experimental systems

n/a	Involved in the study
☐	☒ Antibodies
☒	☐ Eukaryotic cell lines
☒	☐ Palaeontology and archaeology
☐	☒ Animals and other organisms
☐	☒ Clinical data
☒	☐ Dual use research of concern
☒	☐ Plants

Methods

n/a	Involved in the study
☒	☐ ChIP-seq
☒	☐ Flow cytometry
☒	☐ MRI-based neuroimaging

Antibodies

Antibodies used

Primary antibodies:
anti-LUM (Abcam, ab168384; clone EPR11380(B); Lot GR121948-4; 1:75), anti-MMP2 (Abcam, ab97779; Lot GR3448382-1; 1:200), anti-α-SMA (Abcam, ab7817; clone 1A4; Lot 1009584-11;1:600), and anti-PDGFA (Santa Cruz Biotechnology, sc-9974; clone E-10; Lot C0222; 1:600), anti-PDPN (BioLegend, 337005; clone NC-08; Lot B360564; 1:75), and anti-CD68 (Cell Signaling Technology, #79594; clone D4B9C; Lot 779594S; 1:50).

Secondary antibodies:
anti-mouse AF594 (ThermoFisher, A11005; Lot 2538976; 1:1000), and anti-rabbit AF488 (ThermoFisher, A11008; Lot 2557379; 1:1000)

Validation

PDGF-A (E-10) mouse monoclonal; Santa Cruz; cat no. sc-9974; IF 1:50 - 1:500
https://www.scbt.com/p/pdgf-a-antibody-e-10
Validated by the company and the following publication: Gupta S, et al. Role of phosphoinositide 3-kinase in the aggressive tumor growth of HT1080 human fibrosarcoma cells. Mol Cell Biol. 2001 Sep;21(17):5846-56. doi: 10.1128/MCB.21.17.5846-5856.2001.

Alexa Fluor® 488 anti-human Podoplanin (NC-08) rat monoclonal; BioLegend; cat no. 337005; FC 1:100
https://www.biolegend.com/fr-fr/products/alexa-fluor-488-anti-human-podoplanin-antibody-5961
Validated by the company and the following publication: Fujino N, et al. A novel method for isolating individual cellular components from the adult human distal lung. Am J Respir Cell Mol Biol. 2012 Apr;46(4):422-30. doi: 10.1165/rcmb.2011-0172OC.

Anti-MMP2 rabbit polyclonal; abcam; cat no. ab97779; IF 1:100 - 1:1000
https://www.abcam.com/en-sg/products/primary-antibodies/mmp2-antibody-ab97779
Validated by the company.

Anti-alpha smooth muscle Actin (1A4); abcam; cat no. ab7817; IF 1 ug/ml
https://www.abcam.com/en-sg/products/primary-antibodies/alpha-smooth-muscle-actin-antibody-1a4-ab7817
Validated by the company and the following publication: Huang L, Zet al. Interferon regulatory factor 7 protects against vascular smooth muscle cell proliferation and neointima formation. J Am Heart Assoc. 2014 Oct 10;3(5):e001309. doi: 10.1161/JAHA.114.001309.

Anti-12 Lipoxygenase/ALOX12 [EPR11380(B)] Rabbit Recombinant Monoclonal; abcam; cat no. ab168384; WB 1:1000 - 1:5000
https://www.abcam.com/en-sg/products/primary-antibodies/12-lipoxygenase-alox12-antibody-epr11380b-ab168384
Validated by the company.

CD68 (D4B9C) (PE Conjugate) rabbit monoclonal; Cell Signaling Technology #79594; FC 1:50
https://www.cellsignal.com/products/antibody-conjugates/cd68-d4b9c-xp-rabbit-mab-pe-conjugate/79594
Validated by the company.

Animals and other research organisms

Policy information about studies involving animals; ARRIVE guidelines recommended for reporting animal research, and Sex and Gender in Research

Laboratory animals

8 weeks old(Female) wild type C57BL/6NTac mice were purchased from InVivos (https://www.invivos.com.sg/) and were sacrificed immediately upon delivery for all the animal experiments in this study.

Wild animals

No wild animals were used in this study.

Reporting on sex

As the goal of this study was to demonstrate a technology, sex is not relevant to this study. Female mice were used in this study.

Field-collected samples

No field-collected samples were used in this study.

Ethics oversight

All animal care and experiments were carried out in accordance with Agency for Science, Technology and Research (A*STAR) Institutional Animal Care and Use Committee (IACUC) guidelines (IACUC #211580).

Note that full information on the approval of the study protocol must also be provided in the manuscript.

Clinical data

Policy information about clinical studies

All manuscripts should comply with the ICMJE guidelines for publication of clinical research and a completed CONSORT checklist must be included with all submissions.

Clinical trial registration	This study is a demonstration of the FISHnCHIPs technology and not part of a clinical trial.
Study protocol	We were not involved in and do not have access to the design of study protocol.
Data collection	We obtained non-identifiable sections of human tissue from our clinical collaborators.
Outcomes	We do not have access to the outcome measures and we did not use such information in our study.
